# A multi-outcome prognostic model for older adults admitted to skilled nursing facilities for hip fracture and stroke

**DOI:** 10.1093/geroni/igaf122.2445

**Published:** 2025-12-31

**Authors:** W James Deardorff, Siqi Gan, Bocheng Jing, John Boscardin, Alex Smith, Sei Lee

**Affiliations:** University of California, San Francisco, San Francisco, California, United States; University of California, San Francisco, San Francisco, California, United States; University of California, San Francisco, San Francisco, California, United States; University of California, San Francisco, San Francisco, California, United States; University of California, San Francisco, San Francisco, California, United States; University of California, San Francisco, San Francisco, California, United States

## Abstract

Many hospitalized older adults are discharged to a skilled nursing facility (SNF) for short-term rehabilitation following a hip/femur fracture or stroke. Mortality rates are high, and many never return home (i.e., high risk of “rehabbed to death”). To inform shared decision-making, we developed prognostic models using a 20% Medicare sample from 2017-2019 of community-dwelling adults aged ≥66 admitted to a SNF for hip/femur fracture or stroke. Within each cohort, we developed a prognostic model to predict 2 outcomes: 6-month mortality and “successful community discharge” (discharge to the community without subsequent rehospitalization or death within 30 days). Model predictors were pre-specified based on literature review: age, sex, hospital length of stay, Medicaid status, comorbidities, and hospitalizations in the past year. Model performance was assessed by discrimination (c-statistic) and calibration (calibration plots). Internal validation was performed via bootstrapping. The hip/femur fracture cohort included 52,843 individuals (mean age 83.4 years, 73% female, 3.3% Black), and the stroke cohort included 20,599 individuals (mean age 81.7 years, 60.1% female, 11.4% Black). Overall, in the hip/femur fracture and stroke cohorts, 15.5% and 24.6% died within 6 months and 60.1% and 45.4% experienced a successful community discharge, respectively. For the hip/femur fracture and stroke cohorts, the optimism-corrected c-statistics were 0.74 and 0.70 for 6-month mortality and 0.68 and 0.67 for successful community discharge, respectively. Calibration plots showed that the models were well-calibrated for both outcomes in both cohorts. Risk predictions from these models can help guide shared decision-making and future planning between SNF clinicians, patients, and caregivers.

